# Sc2Mol: a scaffold-based two-step molecule generator with variational autoencoder and transformer

**DOI:** 10.1093/bioinformatics/btac814

**Published:** 2022-12-28

**Authors:** Zhirui Liao, Lei Xie, Hiroshi Mamitsuka, Shanfeng Zhu

**Affiliations:** School of Computer Science, Fudan University, Shanghai 200433, China; Department of Computer Science, Hunter College, The City University of New York, New York, NY 10065, USA; Bioinformatics Center, Institute for Chemical Research, Kyoto University, Uji, Kyoto Prefecture 611-0011, Japan; Department of Computer Science, Aalto University, Espoo 00076, Finland; Institute of Science and Technology for Brain-Inspired Intelligence and MOE Frontiers Center for Brain Science, Fudan University, Shanghai 200433, China; Shanghai Qi Zhi Institute, Shanghai 200030, China; Key Laboratory of Computational Neuroscience and Brain-Inspired Intelligence, Fudan University, Ministry of Education, Shanghai 200433, China; Shanghai Key Lab of Intelligent Information Processing and Shanghai Institute of Artificial Intelligence Algorithm, Fudan University, Shanghai 200433, China; Zhangjiang Fudan International Innovation Center, Shanghai 200433, China; Institute of Artificial Intelligence Biomedicine, Nanjing University, Nanjing, Jiangsu 210031, China

## Abstract

**Motivation:**

Finding molecules with desired pharmaceutical properties is crucial in drug discovery. Generative models can be an efficient tool to find desired molecules through the distribution learned by the model to approximate given training data. Existing generative models (i) do not consider backbone structures (scaffolds), resulting in inefficiency or (ii) need prior patterns for scaffolds, causing bias. Scaffolds are reasonable to use, and it is imperative to design a generative model without any prior scaffold patterns.

**Results:**

We propose a generative model-based molecule generator, Sc2Mol, without any prior scaffold patterns. Sc2Mol uses SMILES strings for molecules. It consists of two steps: scaffold generation and scaffold decoration, which are carried out by a variational autoencoder and a transformer, respectively. The two steps are powerful for implementing random molecule generation and scaffold optimization. Our empirical evaluation using drug-like molecule datasets confirmed the success of our model in distribution learning and molecule optimization. Also, our model could automatically learn the rules to transform coarse scaffolds into sophisticated drug candidates. These rules were consistent with those for current lead optimization.

**Availability and implementation:**

The code is available at https://github.com/zhiruiliao/Sc2Mol.

**Supplementary information:**

Supplementary data are available at *Bioinformatics* online.

## 1 Introduction

In drug discovery, researchers aim to find molecules with desired pharmaceutical properties. However, due to permutations of atoms and bonds, the chemical space is huge: the number of potential drug-like (synthesizable) molecules is estimated to be more than 10^23^ (Polishchuk *et al.*, 2013), which is an intractable number for exhaustive search by wet-lab experiments. Deep learning-based generative models, which learn the probability distribution of a given massive training dataset and then have succeeded in generating objects, such as images (Arjovsky *et al.*, 2017; Karras *et al.*, 2020), text (Zhang *et al.*, 2017, 2019) and music (Dong *et al.*, 2018) from the learned distribution, will thus be useful for searching drug candidates in the huge chemical space.

Molecule generation, which usually represents a molecule by a SMILES string (Weininger, 1988) or a molecular graph, has two tasks: distribution learning and molecule optimization (Langevin *et al.*, 2020). The first is that a distribution is modeled from a given training dataset, and novel molecules with properties similar to the training dataset are generated from the distribution by random sampling. The second is to modify input molecules and generate molecules with improved scores according to the given evaluation function. Usually, molecules are generated from atoms and bonds in a *de novo* style (e.g. Blaschke *et al.*, 2020; Gómez-Bombarelli *et al.*, 2018). Nonetheless, each molecule has a backbone structure called a *scaffold*. A suitable scaffold is necessary for a molecule to match the binding pocket of a target protein. In addition, synthesizing organic compounds from intermediates with the same scaffolds can reduce medicinal chemistry efforts. Therefore, generating a molecule through a scaffold is a common and efficient practice in drug discovery (Zhang *et al.*, 2007). However, all recent scaffold-based approaches need expert knowledge, such as pre-defined patterns (Arús-Pous *et al.*, 2020; Langevin *et al.*, 2020; Li *et al.*, 2020; Lim *et al.*, 2020). Moreover, in these approaches, a scaffold is defined as a fragment or a substructure (rather than a backbone), being likely to generate molecules with larger shapes than input scaffolds, regardless that a moderate size is important for protein–drug binding. Also, clinical drug candidates should be generated from not only known compounds but also large compound collection through random screening (Brown and Boström, 2018). Thus, molecule generation should support both random *de novo* generation and lead molecule optimization.

**Fig. 1.**
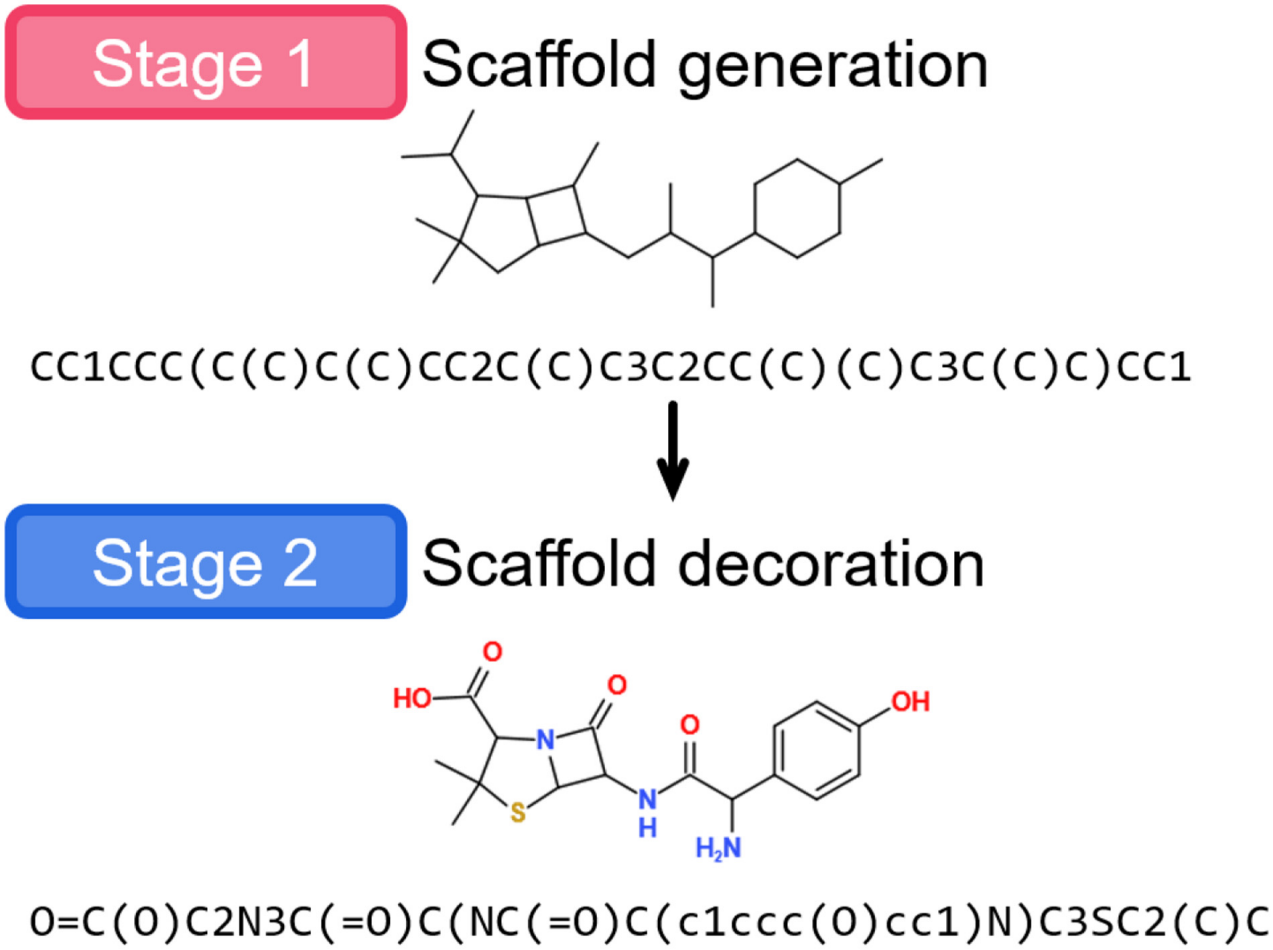
Two-step molecule generation: we first generate a scaffold that only contains carbon atoms and single bonds and then decorate this scaffold with different atoms and bonds

We propose an end-to-end deep generative model, Sc2Mol, for generating molecules (represented by SMILE strings) with two steps: scaffold generation and scaffold decoration. We first generate a scaffold that contains only carbon atoms and single bonds by a variational autoencoder (VAE) (Kingma and Welling, 2014), which provides a scaffold distribution to find a novel scaffold. We then enrich the generated scaffold by changing atom and bond types by a transformer (Vaswani *et al.*, 2017), resulting in molecules with desired properties (see Fig. 1). Our model needs no extra expert knowledge, such as grammar rules and pre-defined substructures, and generates molecules from either random variables or given scaffolds.

We used the MOSES dataset (Polykovskiy *et al.*, 2020) and a subset of the ZINC database (Gómez-Bombarelli *et al.*, 2018; Sterling and Irwin, 2015) (both of them are drug-like molecule datasets) to evaluate our model by comparing with well-known baselines: CharVAE (Gómez-Bombarelli *et al.*, 2018), JTVAE (Jin *et al.*, 2018) and MoFlow (Zang and Wang, 2020). Our model achieved comparable performances against the competing methods on randomly generating molecules under several evaluation metrics. The results showed that our model, even though without pre-defined rules, could capture complex SMILES syntax, including matching parentheses for side chains and pairing numbers for ring systems and lower cases for aromatic systems, and chemical rules, such as the similarity between halogens. On the other hand, given scaffolds, Sc2Mol generated molecules by carefully considering the trade-off between the scaffold and molecule similarity. That is, keeping similarity to a reference molecule, our model could reduce the scaffold similarity, even when the scaffolds are not in the training set. Finally, several case studies demonstrated that our model could convert simple carbon scaffolds into potential drug-like compounds.

Our contribution can be summarized into the following three points:

- We present a SMILES-based deep generative model called Sc2Mol for molecule generation, which consists of two steps: scaffold generation and scaffold decoration.
- Sc2Mol does not need any expert knowledge, such as pre-defined patterns or syntactic rules. It allows both random *de novo* molecule generation and scaffold transformation (to a molecule with desired properties).
- Our experimental results showed that Sc2Mol could learn chemical rules and patterns automatically and could discover potential compounds for mental illness by using the learned rules and patterns.

## 2 Materials and methods

### 2.1 Problem formulation

We use a string (SMILES; Weininger, 1988) to represent a molecule, meaning that molecule generation can be a text generation problem. Thus, our problem is, given a source string with *l* characters, $x=(x_{1},x_{2},\ldots ,x_{l})$, to generate a target string with $l^{\prime }$ characters, $y=(y_{1},y_{2},\ldots ,y_{l^{\prime }})$. We assume that the source and target strings share the same vocabulary with the size of *v*.

**Fig. 2.**
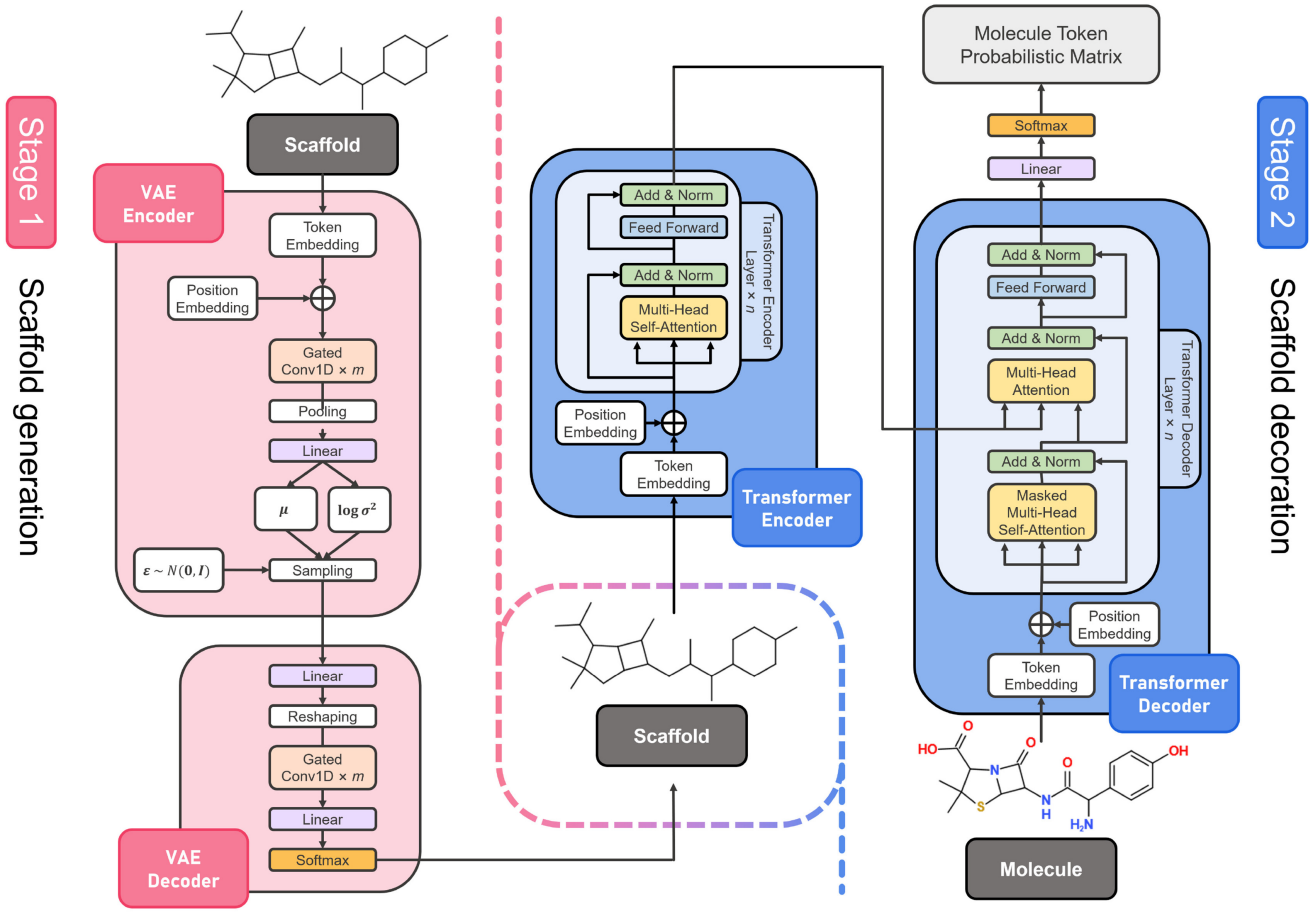
Our model architecture. For scaffold generation, we use a VAE that receives the source scaffold, keeping the balance between the reconstruction error and the KL-divergence. For scaffold decoration, we use a transformer to transform the generated carbon scaffold into a meaningful molecule string, close to the target molecule string

Our method for this problem has two steps (Fig. 1): (i) scaffold generation: generating a *generic* scaffold and (ii) scaffold decoration: decorating the generic scaffold with atoms and bonds. A generic scaffold can be defined, following (Blaschke *et al.*, 2020): a generic (carbon) scaffold is a molecule obtained by replacing all types of non-hydrogen atoms by carbon atoms and all types of bonds by single bonds. Note that a generic scaffold is also a valid molecule. This definition can keep the original molecule shape as possible. We use a VAE for scaffold generation (i.e. a string generation problem) and a transformer for scaffold decoration corresponding to translation from a generic scaffold string to the desired molecule string. Figure 2 shows our entire architecture.

### 2.2 Scaffold generation

We assume that the latent prior distribution is a standard normal distribution. We represent a character by a one-hot row vector; thus, an input SMILES string with *l* characters can be denoted by a binary matrix $X\in R^{l\times v}$. We first apply an embedding layer with a positional encoding to ***X***, to learn dense representation from the input. Note that the lookup operation on an embedding matrix can be considered as matrix multiplication, resulting in the following update for the embedding layer:

$$X_{1}=XE_{1}+P_{1},$$

where $X_{1}\in R^{l\times d}$ is the embedding result, $E_{1}\in R^{v\times d}$ is an embedding matrix, and $P_{1}\in R^{l\times d}$ is a positional encoding matrix.

We implement a VAE by stacking *m* gated convolutional neural networks (GatedConv) for strings (Dauphin *et al.*, 2017) with residual connection (He *et al.*, 2016):

$$\begin{array}{c} X_{t+1}=X_{t}+\text{GatedConv}(X_{t}),\;t=1,2,\ldots ,m,\\ \;\text{where}\;\text{GatedConv}(A)=(A⋆U_{1}+c_{1})⊗s(A⋆U_{2}+c_{2}), \end{array}$$

⋆ and ⊗ denote the convolution operation and the element-wise product, respectively, *s* is the sigmoid function, $U_{1},U_{2}\in R^{k\times d\times d}$ are convolution kernels of size *k*, and $c_{1},c_{2}\in R^{d}$ are biases. We then use max pooling to reduce the convolution result to vector $h\in R^{d}$, to obtain mean $\mu \in R^{d_{z}}$ and logarithmic variance $\text{log}\;\sigma \in R^{d_{z}}$ of the approximate distribution by two independent fully connected layers:

$$\begin{array}{c} h=\text{MaxPooling}(X_{m+1}).\\ \mu =hW_{1}+b_{1},\;\;\;\;\text{log}\;\sigma^{2}=hW_{2}+b_{2}. \end{array}$$

Note that direct sampling from N(μ,σ) is non-differentiable with respect to μ and σ, and thus we apply the reparameterization trick below:

z=μ+σ⊗ϵ, where ϵ∼N(0,I).

From the obtained latent variable ***z***, we reconstruct the input SMILES string using the following decoder:

$$\begin{array}{c} Z_{1}=\text{Reshape}(z^{\prime }),\;\;\text{where}\;\;z^{\prime }=zW_{3}+b_{3}.\\ Z_{t+1}=Z_{t}+\text{GatedConv}1D(Z_{t}),\;t=1,2,\ldots ,m.\\ \hat{X}=\text{Softmax}(Z_{m+1}W_{4}+b_{4}). \end{array}$$

This VAE is designed to model generic carbon scaffolds in a latent space of dimension $d_{z}$, and then sample a random variable from this space to construct a certain scaffold, entirely allowing scaffold hopping.

### 2.3 Scaffold decoration

We decorate each carbon scaffold (output of the VAE) by using a transformer (Vaswani *et al.*, 2017) to generate the desired compound. Note that the carbon scaffold is a probabilistic matrix rather than a one-hot index matrix, and the argmax operation is non-differentiable. Thus, for training, we use ‘teacher forcing’ (Williams and Zipser, 1989), i.e. the ground truth scaffold, to avoid the non-differentiable argmax operation, while for inference, we directly use the scaffold generated by the VAE through the argmax and one-hot operation:

$$X^{\prime }=\tilde{X}E_{2}+P_{2},\;\text{where}\;\;\tilde{X}=\{\begin{array}{cc} X & \text{for training};\\ \text{argmax}(\hat{X}) & \text{for inference}. \end{array}$$

$X^{\prime }\in R^{l\times d}$ is the embedding result of the input scaffold; $E_{2}\in R^{v\times d}$ and $P_{2}\in R^{l\times d}$ are the token embedding matrix and positional encoding matrix, respectively, of the transformer. We combine multi-head attention with residual connection (He *et al.*, 2016), layer normalization (Lei Ba *et al.*, 2016) and feed-forward networks to assemble the encoder and decoder layers in the transformer. The decorated SMILES string $\hat{Y}$ is given by a fully connected layer with the softmax activation.

### 2.4 End-to-end style training of the VAE and transformer

Assuming that prior distribution $p_{\theta }(z)$ is a standard normal distribution, the VAE loss function can be given as follows:

(1) $$\begin{array}{c} L_{\mathrm{vae}}=\gamma \cdot L_{KL}+L_{vr},\;\;\text{where}\\ L_{KL}=D_{KL}(N(\mu ,\sigma^{2}I)||N(0,I))=-\frac{1}{2}\sum_{i=1}^{d}\left(1+\text{log}\;\sigma_{i}^{2}-\mu_{i}^{2}-\sigma_{i}^{2}\right)\\ \text{and}\;\;L_{vr}=\text{CrossEntropy}(X,\hat{X})=-\frac{1}{l}\sum_{j=1}^{l}\sum_{k=1}^{v}x_{jk}\;\text{log}\;{\hat{x}}_{jk} \end{array}$$

The VAE loss function (1) balances between the KL-divergence and the reconstruction error by *γ*, the weight over the KL-divergence:

$$\gamma =\text{min}(0.01,0.001+\frac{\left\lfloor \text{max}(0,\mathrm{step}\_\mathrm{num}-40000)/5000\right\rfloor }{10000}).$$

Note that this manner has been adopted to improve training for sentence generation (Bowman *et al.*, 2016).

For training the transformer, we use the cross entropy loss function:

$$L_{tr}=\text{CrossEntropy}(Y,\hat{Y})=-\frac{1}{l^{\prime }}\sum_{j=1}^{l^{\prime }}\sum_{k=1}^{v}y_{jk}\;\text{log}\;{\hat{y}}_{jk},$$

where ***Y*** is the one-hot matrix of the target molecule string and $\hat{Y}$ is the output probabilistic matrix.

Finally, the total loss function is:

$$L=\gamma \cdot L_{KL}+L_{vr}+L_{tr}.$$

For optimization, we use the Adam optimizer (Kingma and Ba, 2015) with the learning rate of the following warm-up schedule (Vaswani *et al.*, 2017):

$$lr=d^{-\frac{1}{2}}\cdot \text{min}(\mathrm{step}\_num^{-\frac{1}{2}},\mathrm{step}\_\mathrm{num}\cdot 10000^{-\frac{3}{2}}).$$

### 2.5 Two types of molecule generation

Our model allows two types of molecule generation (i) from a random latent variable and (ii) from a given scaffold, which are *de novo* molecule generation and molecule optimization, respectively. The first type randomly samples a variable from the standard normal distribution and uses the decoder of the VAE and transformer to obtain a molecule without any expert knowledge. The second type starts with an input carbon scaffold and obtains a molecule with proper atoms and bonds based on this scaffold.

To improve the validity of generated molecules, we added a validity-check component to our model for inference. This component will check the validity of output strings. If the output string is invalid according to the SMILES syntax, the model will discard the string and attempt to make a new generation.

## 3 Experiments

### 3.1 Dataset

We used two datasets to evaluate model performance. The first one is the MOSES dataset (Polykovskiy *et al.*, 2020), derived by filtering from ZINC (Sterling and Irwin, 2015). In MOSES, one molecule has a molecular weight ranging from 250 to 350 Da, no charged atoms, and no rings larger than eight atoms, and atom types are limited to H, C, N, O, F, S, Cl and Br only. Also, all molecules are drug-like, since they pass the medicinal chemistry filters and PAINS filters (Baell and Holloway, 2010). MOSES consists of three subsets: training set, test set and novel scaffold set, with around 1.6 million, 176 000 and 176 000 molecules, respectively. All scaffolds in the novel scaffold set differ from those in both the train and test sets.

The second one is the ZINC-250k dataset (Gómez-Bombarelli *et al.*, 2018), which was built by randomly extracting about 250 000 drug-like molecules from ZINC (Sterling and Irwin, 2015). A molecule in this dataset is commercially available and has no rings larger than eight atoms, and atom types are limited to H, C, N, O, F, P, S, Cl, Br and I only. We randomly split the ZINC-250k into train (80%) and test (20%) sets.

We trained our model by the training set, and the trained model was evaluated by the following three tasks: Task (1) random generation from latent variables; Task (2) scaffold decoration for the test set molecules, keeping input as generic scaffold molecules; Task (3) scaffold decoration for the novel scaffold set, keeping the same as the above (2).

### 3.2 Experiment setting

We set model dimension *d* as 256 and latent dimension *d_z_* as 64; both the VAE and transformer had a three-layer encoder and a three-layer decoder. All gated convolution layers in the VAE had a kernel size of 3 with a stride length of 1. The transformer used four-head multi-head attention and feed-forward dimension *d_ff_* was set at 1024. We set the batch size to 64, and adopted the Adam optimizer (Kingma and Ba, 2015) with the learning rate schedule shown in Equation (2). To reduce over-fitting, a drop-out rate (Srivastava *et al.*, 2014) of 0.1 was applied. Hyperparameter settings and selection can be found in Supplementary Tables S1–S3.

We used RDKit (https://www.rdkit.org/) for data preprocessing and implemented our model by Tensorflow (https://www.tensorflow.org/) on a machine with NVIDIA GeForce GTX 1080 Ti GPU.

### 3.3 Baselines

Table 1 is a comparison of baseline models (and our model) shown below:

**Table 1.** Comparison of models

Model	Molecular representation	Random generation	Generation from scaffold	Need pre-defined objects	Model architecture
AddCarbon	SMILES	No	Yes	No	—
CharVAE	SMILES	Yes	Yes	No	VAE with RNN
FragLinker	SMILES	No	Yes	Yes	Transformer
JTVAE	Graph	Yes	Yes	Yes	VAE with RNN
MoFlow	Graph	Yes	Yes	No	GCN + CNN
Sc2Mol(Ours)	SMILES	Yes	Yes	No	VAE with CNN + Transformer

- AddCarbon (Renz *et al.*, 2019): a simple model that adds a carbon token ‘C’ to the source SMILES string at a random position.
- CharVAE (Gómez-Bombarelli *et al.*, 2018): has a VAE for SMILES strings, with convolution layers, followed by a fully connected layer, and a decoder with gated recurrent unit networks (Chung *et al.*, 2014).
- FragLinker: a SMILES-based model, an extended variant of SyntaLinker (Yang *et al.*, 2020). The original SyntaLinker receives only two fragment strings as input and uses a transformer to link the input into a completed molecule. This input is extended into any fragment strings.
- JTVAE (Jin *et al.*, 2018): first decomposes a molecule into a junction tree, where each node refers to a pre-defined subgraph, and then uses a VAE, to encode both the junction tree and subgraphs into two latent variables, which are decoded to reconstruct the junction tree and assemble subgraphs according to the tree.
- MoFlow (Zang and Wang, 2020): a graph-based flow model that uses two invertible neural networks to encode atoms and bonds into two Gaussian latent variables and then uses the reverse neural networks to transform Gaussian noise variables into atoms and bonds. This model adopts a validity correction module to ensure chemical validity.

### 3.4 Evaluation measures

All models were trained with the training sets and tested with the test or novel scaffold sets for evaluation. Baseline models were trained in their originally designed ways, in which inputs and expected outputs were molecules.

For Task 1, we randomly drew 30 000 samples from the standard normal distribution which are the input of the decoder of the variational autoencoder and the rest of the model. For Tasks 2 and 3, we randomly selected 30 000 molecules from the test/novel scaffold set and extracted the carbon scaffolds, which are then the input of the whole model. We first adopted the three common metrics of molecule generation:

- **Validity**: The ratio of the chemically valid molecules to the totally generated molecules. Higher validity means that the model learns more correctly proper chemical rules, such as valence and aromaticity.
- **Uniqueness**: The ratio of the uniquely generated valid molecules to the totally generated valid molecules. Higher uniqueness indicates that the model can generate more diverse molecules.
- **Novelty**: The ratio of the novel valid generated molecules (not in the training set) to the totally valid generated molecules. High novelty means that the model generates molecules not in the training set more.

Also, we checked the distribution of molecular weights, calculated octanol-water partition coefficients (logP) (Wildman and Crippen, 1999) and quantitative estimates of drug-likeness (QED) (Bickerton *et al.*, 2012) to illustrate the similarity of the generated molecules to the training set (also the test set for Task 2 and the novel scaffold set for Task 3).

Additionally, for Tasks 2 and 3, we introduced the following metrics:

- **Recovery**: The ratio of the desired valid generated molecules (identical to the corresponding reference molecules in the test set) to the totally valid generated molecules. Recovery is proportional to how well the model learned the optimization rules from a scaffold to the desired molecule.
- **Similarity**: The average Tanimoto similarity between fingerprints [1024-bit extended-connectivity fingerprints with radius 2 (ECFP4) (Rogers and Hahn, 2010)] of generated molecules and the corresponding reference molecules in the test set. Similarity is proportional to how well the model captures the optimization rules.
- **Scaffold similarity (SS)**: The average Tanimoto similarity between fingerprints of the scaffold of generated molecules and the scaffold of the corresponding reference molecules in the test set. Moderate SS is favorable since high SS implies no novel scaffolds, and low SS implies arbitrary generation without considering input scaffolds.

### 3.5 Results

#### 3.5.1 Task 1: Random generation

Table 2 shows the performance on the MOSES dataset (three common metrics) of baselines and our model (note that AddCarbon and FragLinker cannot be applied to Task 1), indicating that our model achieved the best in Uniqueness and Novelty. JTVAE achieved the Validity of 100% because of tree decomposition and pre-defined subgraphs, avoiding learning SMILES syntax. MoFlow and our model also achieved the Validity of 100% as well because of benefiting from the validity-check component. Supplementary Table S4 shows the performance on the ZINC-250k dataset. Similar to the case on the MOSES dataset, our model still achieved the best in three metrics.

**Table 2.** Performances of models for Task 1 on the MOSES dataset

Model	Validity^a^ (%)	Uniqueness^a^ (%)	Novelty^a^ (%)
AddCarbon	—	—	—
CharVAE	3.33	86.59	99.50
FragLinker	—	—	—
JTVAE	**100.00**	99.92	95.98
MoFlow	**100.00**	99.59	99.61
Sc2Mol (Ours)	**100.00**	**99.99**	**99.72**

a Higher is better.

*Note*: The best results are highlighted in bold.

**Fig. 3.**
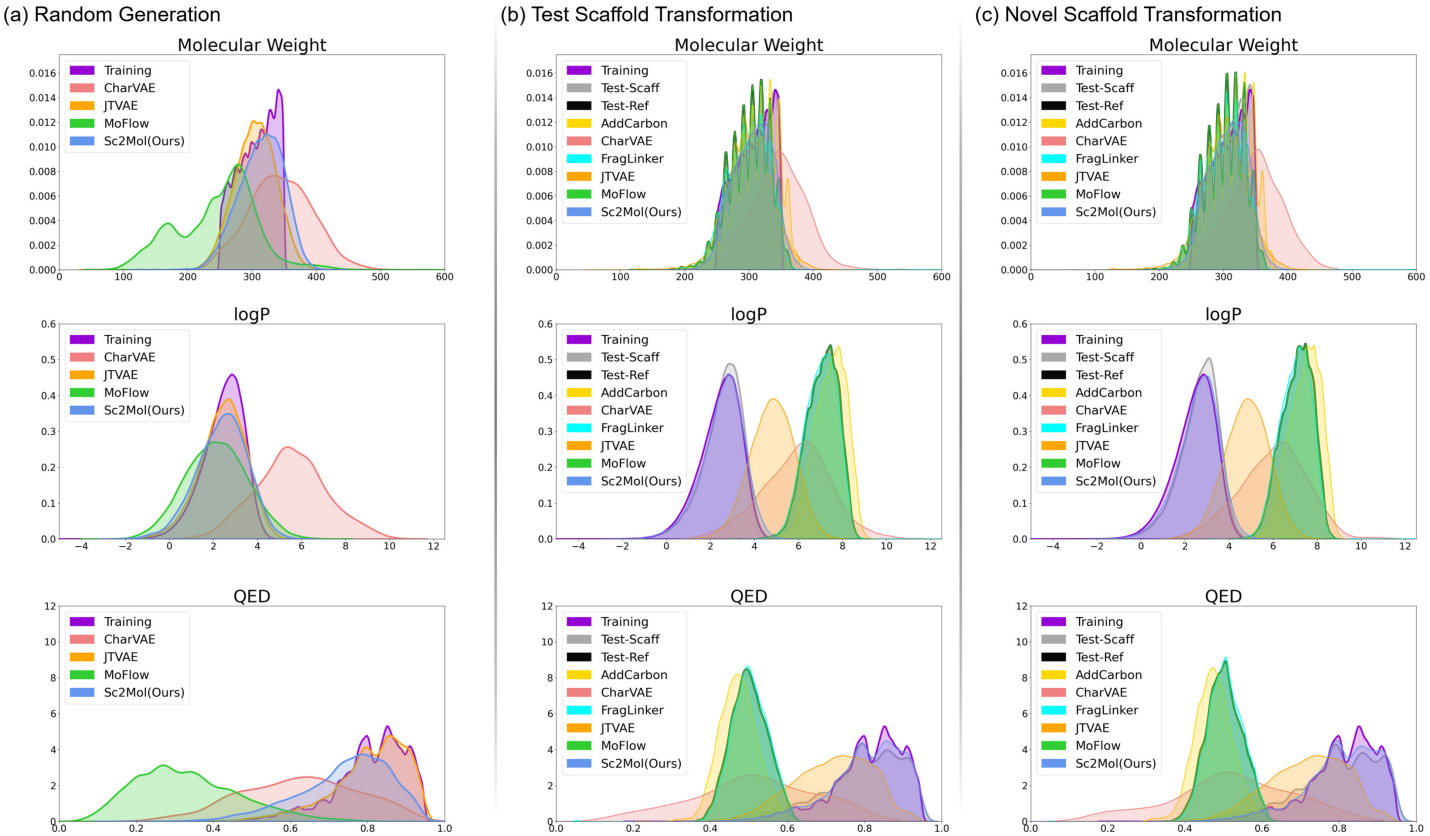
Distributions of the properties (molecular weights, logP and QED) of the generated molecules in Tasks (**a**) 1, (**b**) 2 and (**c**) 3

Figure 3a shows the distributions of molecular weights, logP and QED of the molecules generated in Task 1. Regarding molecular weights and logP, JTVAE and our model showed distributions closer to the training set. Regarding QED (which can be affected by more physical features, such as the molecular polar surface area of molecules), the distribution of JTVAE was the most similar to the training set due to the pre-defined subgraph vocabulary of JTVAE. Without any prior knowledge, our model made the distribution slightly deviate from the training set, and CharVAE made it more away. Most molecules generated by MoFlow had significantly lower QED, whose distribution was far away from the training set.

#### 3.5.2 Task 2: Test scaffold transformation

Table 3 shows the performance in six metrics of all models. AddCarbon, FragLinker and MoFlow achieved good scores (>70%) of Validity, Uniqueness and Novelty, but low Recovery (0.00%) and low Similarity (<10%) with high SS (>80%). It means that these models could not modify scaffolds well enough. Particularly, MoFlow showed 100% SS, indicating it was a perfect autoencoder but not a good modifier for scaffold transformation. JTVAE also achieved high scores (>90%) of Validity, Uniqueness and Novelty, while CharVAE performed clearly worse. These two models showed low Recovery (0.00%), Similarity (<10%) and SS (<30%), implying that they could not pay enough attention to the input scaffolds. By contrast, our model achieved the best Validity, Uniqueness and Similarity, and it even recovered some (4.93%) desired reference molecules, although its Novelty decreased. The SS of our model also decreased to a moderate value (72.94%), suggesting that our model performed the best overall.

**Table 3.** Performance of models on Task 2 (generating molecules from test scaffolds)

Model	Validity^a^ (%)	Uniqueness^a^ (%)	Novelty^a^ (%)	Recovery^a^ (%)	Similarity^a^ (%)	Scaffold similarity (SS)^b^ (%)
AddCarbon	99.94	97.56	**100.00**	0.00	6.07	81.04
CharVAE	1.88	78.90	**100.00**	0.00	11.23	24.69
FragLinker	95.84	87.33	**100.00**	0.00	6.10	98.79
JTVAE	99.48	93.77	99.99	0.00	8.69	24.51
MoFlow	**100.00**	88.48	**100.00**	0.00	6.17	100.00
Sc2Mol (Ours)	**100.00**	**97.58**	80.23	**4.93**	**36.37**	72.94

a Higher is better.

b An appropriate value is good.

*Note*: The best results are highlighted in bold.

Figure 3b shows the distributions of molecular weights, logP and QED of the molecules generated in Task 2. The distributions of molecular weights were all rather similar to each other, except CharVAE. For logP and QED, the distributions of CharVAE and JTVAE had no significant overlaps with other distributions. However, those of AddCarbon, FragLinker and MoFlow were almost identical to that of the scaffolds of the test set. More promisingly, the distributions of our model were extremely similar to those of the training set and the (references of the) test set.

In summary, (i) CharVAE and JTVAE generated molecules without careful attention to the input scaffolds, resulting in low Similarity, low SS and little overlap with other distributions. (ii) AddCarbon, FragLinker and MoFlow, which modified input scaffolds only slightly, were unable to transform the carbon scaffolds into desired molecules, resulting in only generating molecules very similar to the input scaffolds. (iii) Our model could achieve the highest molecular similarity to the reference molecules and the distribution most highly similar to those molecules. Also, the SS values implied the possibility of generating the most desired scaffolds.

#### 3.5.3 Task 3: Novel scaffold transformation

Table 4 shows the performance of all models under the six metrics, and Figure 3c shows the distributions of the generated molecules by all models. Entirely the results are consistent with those for Task 2. Note that our model could generate molecules most similar to the reference molecules, regardless that the input scaffolds are not explicitly in the training set. This result confirms that our model can capture, from the given data, rules of transforming scaffolds into desired molecules and apply the rules to even data with unseen scaffolds successfully.

**Table 4.** Performance of models on Task 3 (generating molecules from novel scaffolds)

Model	Validity^a^ (%)	Uniqueness^a^ (%)	Novelty^a^ (%)	Recovery^a^ (%)	Similarity^a^ (%)	Scaffold similarity (SS) (%)^b^
AddCarbon	**99.96**	94.94	**100.00**	0.00	5.98	81.34
CharVAE	1.79	78.21	**100.00**	0.00	11.05	24.39
FragLinker	95.74	74.71	**100.00**	0.00	6.05	98.56
JTVAE	99.71	94.04	99.99	0.00	8.65	24.59
MoFlow	**100.00**	76.06	**100.00**	0.00	6.06	100.00
Sc2Mol (Ours)	**100.00**	**95.19**	81.19	**4.11**	**34.53**	71.95

a Higher is better.

b An appropriate value is good.

*Note*: The best results are highlighted in bold.

#### 3.5.4 Ablation study

We also conducted the ablation study to verify the effectiveness of our model with the following experimental settings: (i) Our proposed model and MoFlow with/without the validity-check component were compared to confirm the effectiveness of this component. (ii) Several baseline models were trained with scaffolds as inputs and molecules as expected outputs, which would be tagged with ‘-s2m’. (iii) Our proposed model was trained with molecules as both source inputs and expected outputs, which would be tagged with ‘-m2m’.Settings 2 and 3 could study how the high-capacity neural networks would affect generation, and whether it would be necessary to decompose the generation process into scaffold and molecule.

Table 5 shows the performance of models with/without the validity-check component for Task 1 on MOSES. With validity-check, our model achieved the best Uniqueness and Novelty, and both models reached the Validity of 100%. Without validity-check, the Validity of MoFlow decreased significantly to 33.94%, while our model outperformed MoFlow with a Validity of 63.07%. This result is notable since our model does not have any prior expert knowledge of SMILES syntax. Supplementary Table S5 shows the experiment results for Task 1 on ZINC, which are consistent with those on MOSES. Without validity-check, the Validity of MoFlow decreased to about 30%, while our model still had a better Validity of more than 50%. Besides, the scores of Uniqueness and Novelty were insensitive to validity-check. Since the cost of SMILES syntax check is low and our model still could achieve an acceptable Validity even without validity-check, introducing validity-check to our model would not increase considerable computation.

**Table 5.** Ablation study for Task 1 on MOSES

Model	Validity^a^ (%)	Uniqueness^a^ (%)	Novelty^a^ (%)
MoFlow	**100.00**	99.59	99.61
MoFlow w.o. VC	33.94	99.41	99.18
Sc2Mol	**100.00**	**99.99**	**99.72**
Sc2Mol w.o. VC	63.07	99.98	98.70

w.o. VC, without validity-check component.

a Higher is better.

*Note*: The best results are highlighted in bold.

Table 6 shows the performance of models with different input and expected output settings for Task 2 on MOSES. The training data of CharVAE-s2m and FragLinker-s2m were identical to our models, while those of CharVAE, FragLinker and Sc2Mol-m2m were the same. **Validity, Uniqueness**: Our model achieved the best Validity due to the validity-check component, and transformer-based FragLinker also showed good performance on Validity. Transformer-based models reached high scores of Uniqueness (>80%), while CharVAE and CharVAE-s2m failed to generate enough valid and unique molecules (because of low Validity or Uniqueness). These results confirm that high-capacity architectures of neural networks are necessary. **Novelty, Recovery, Similarity and SS:** Transformer-based models trained with ‘s2m’ data showed lower Novelty but higher Recovery and Similarity than their corresponding version of ‘m2m’, respectively. This means that taking scaffolds as input training data could force models to generate expected molecules, although it would decrease some novelty. Note that simply repeating a few unseen molecules would also achieve very high Novelty, but these molecules would not be desired (with low Similarity to the reference). Thus, molecules with acceptable Novelty and enough Similarity would be preferred. In addition, compared with FragLinker-s2m, our model had higher Similarity but lower SS. This result indicates that decomposing generation into two-step and using VAE for scaffold generation could contribute to finding desired molecules with novel scaffolds. Table 7 shows the performance of the ablation study for Task 3 on MOSES. These experimental results are similar to those for Task 2, indicating the generalization capabilities of models.

**Table 6.** Ablation study for Task 2 on MOSES (generating molecules from test scaffolds)

Model	Validity^a^ (%)	Uniqueness^a^ (%)	Novelty^a^ (%)	Recovery^a^ (%)	Similarity^a^ (%)	Scaffold similarity (SS)^b^ (%)
CharVAE-m2m	1.88	78.90	**100.00**	0.00	11.23	24.69
CharVAE-s2m	12.71	0.21	**100.00**	0.00	6.45	25.87
FragLinker-m2m	95.84	87.33	**100.00**	0.00	6.10	98.79
FragLinker-s2m	95.87	96.27	70.30	1.75	26.87	90.81
Sc2Mol-m2m	**100**	96.89	**100.00**	0.00	6.98	26.53
Sc2Mol-s2m	**100**	**97.58**	80.23	**4.93**	**36.37**	72.94

a Higher is better.

b An appropriate value is good.

*Note*: The best results are highlighted in bold.

**Table 7.** Ablation study for Task 3 on MOSES (generating molecules from novel scaffolds)

Model	Validity^a^ (%)	Uniqueness^a^ (%)	Novelty^a^ (%)	Recovery^a^ (%)	Similarity^a^ (%)	Scaffold similarity (SS)^b^ (%)
CharVAE-m2m	1.79	78.21	**100.00**	0.00	11.05	24.39
CharVAE-s2m	12.65	0.20	**100.00**	0.00	6.52	26.89
FragLinker-m2m	95.74	74.71	**100.00**	0.00	6.05	98.56
FragLinker-s2m	95.84	95.93	68.98	1.67	27.43	90.01
Sc2Mol-m2m	**100.00**	94.78	**100.00**	0.00	7.10	27.35
Sc2Mol-s2m	**100.00**	**95.19**	81.19	**4.11**	**34.53**	71.95

a Higher is better.

b An appropriate value is good.

*Note*: The best results are highlighted in bold.

#### 3.5.5 Examples of the generated molecules

**Fig. 4.**
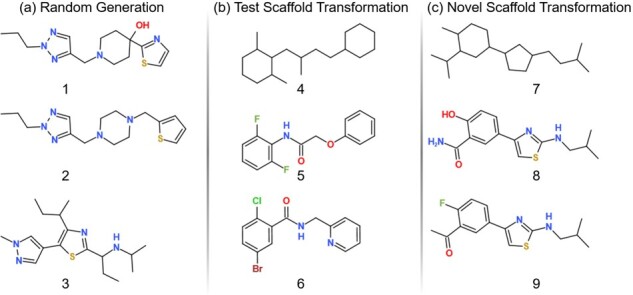
Examples of the generated molecules: (**a**) Task 1: 1–3 from different latent variables, (**b**) Task 2: input scaffold (4), reference (5) and corresponding generated molecule (6), (**c**) Task 3: input scaffold (7), reference (8) and corresponding generated molecule (9)

Figure 4 shows examples of the generated molecules: Figure 4a shows three molecules generated from different latent variables from Task 1. In terms of scaffolds, 1 and 2 were similar while 3 was different, since the corresponding latent variables of 1 and 2 were closer to each other, and far away from that of 3; Figure 4b shows, from Task 2, an input scaffold (4), the reference molecule (5) and the corresponding generated molecule (6) from 4, showing the similarity between 5 and 6, such as the benzene ring and carboxamide. The fluorine at the benzene ring in 5 is replaced by the chlorine in 6. Fluorine and chlorine are in the same group called ‘halogen’, implying that our model was able to discover novel scaffold and learn chemical rules such as halogen similarity and aromatic ring. Figure 4c shows, from Task 3, an input scaffold (7), the reference molecule (8) and the corresponding generated molecule (9). Two molecules, 8 and 9, share the same scaffolds and moieties. The hydroxy group at the benzene ring in 8 is replaced by the fluorine in 9, where fluorinating is a major strategy of lead compound optimization (Brown and Boström, 2018; Young and Leeson, 2018). Also, 8 and 9 have aromatic rings, especially not only benzene but also thiazole. The aromatic rings are special in SMILES, such as lowercase letters for aromatic atoms and a pair of numbers for the starting and end of the ring, implying that our transformer architecture is powerful enough to learn the complex SMILES syntax of aromatic systems.

#### 3.5.6 Case study 1: Auglurant

**Fig. 5.**
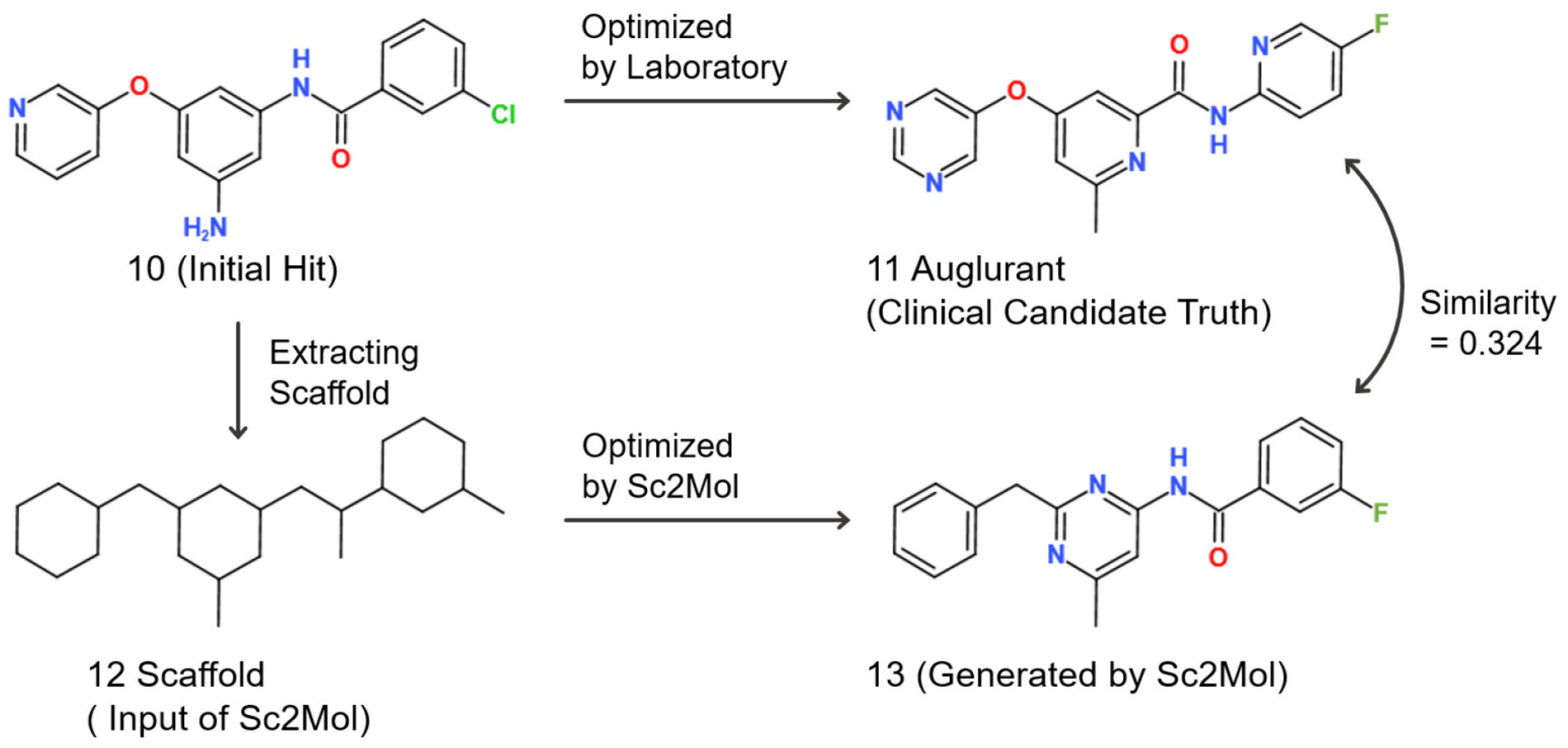
Case study 1, 10: random initial compound for mood disorder target mGluR5, 11: optimized clinical candidate, Auglurant, 12: scaffold from 10 and 13: generated molecule

Figure 5 shows an example prediction by our model, starting with a random screened initial compound (10) [which is, in reality, optimized as Auglurant (11), a clinical candidate for mood disorders whose target is mGluR5 (Bates *et al.*, 2014; Felts *et al.*, 2017)]. The scaffold (12) of 11 was an input of our model, which generated a molecule (13). Note that 11 (ground truth) and 13 (prediction) share the (i) fluorinated benzene, (ii) nitrogen heterocycles and (iii) replacement of the amino group with the methyl group. Importantly, these three points are favorable strategies in lead compound optimization (Brown and Boström, 2018; Pennington and Moustakas, 2017; Young and Leeson, 2018).

#### 3.5.7 Case study 2: Benzodiazepines

**Fig. 6.**
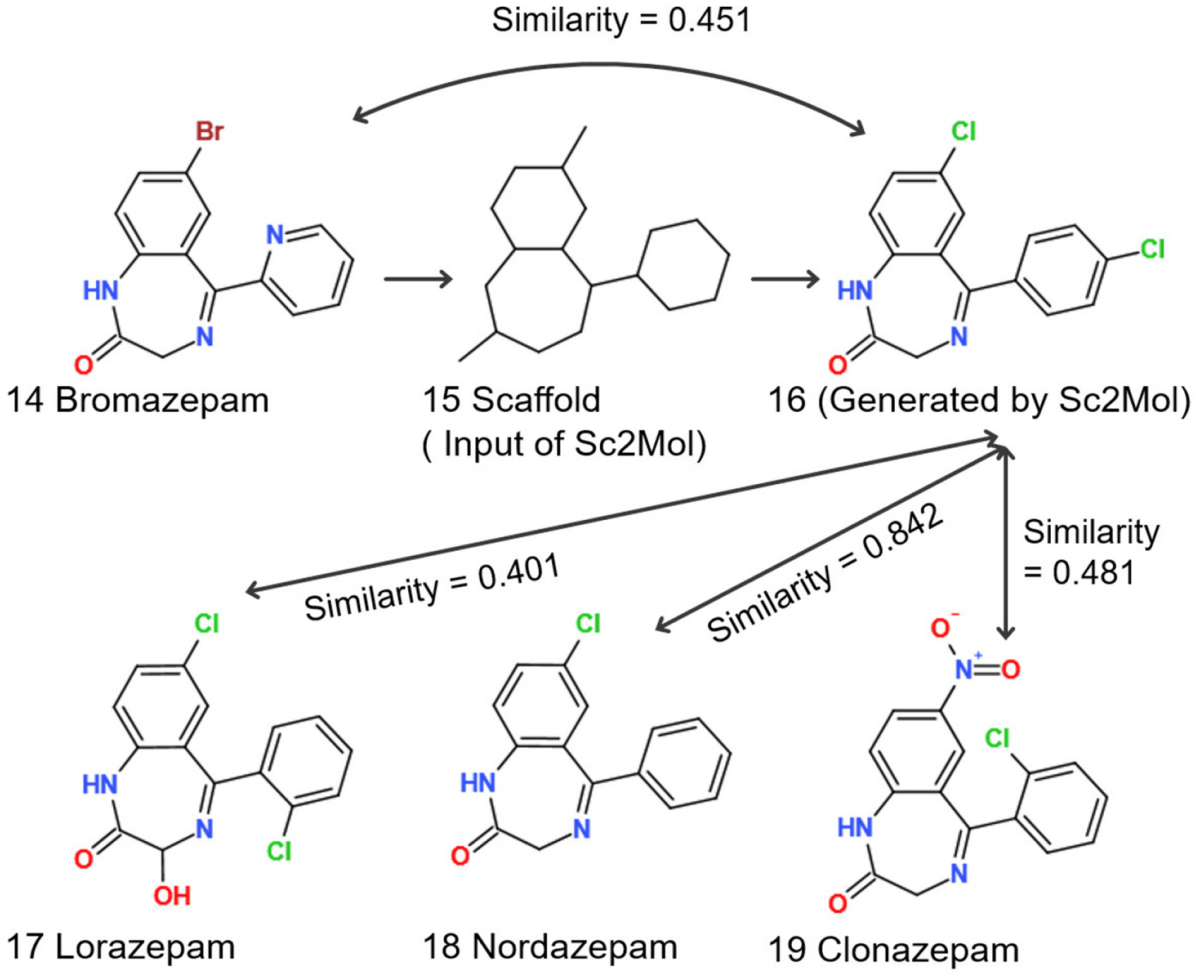
Case study 2 (benzodiazepine), 14: bromazepam, 15: scaffold from 14, 16: generated molecule, 17: lorazepam, 18: nordazepam and 19: clonazepam

Benzodiazepines (BZD) are a category of psychoactive drugs which has a benzene with a diazepine as the core chemical structure. Many compounds in this category reduce brain activity and thus are used to ease mental problems, such as anxiety, insomnia and seizures. Using a training set and additional 46 commercial BZD drugs (Supplementary Table S6), we generated scaffolds first and then molecules. Figure 6 shows our process, starting with bromasepam, an anti-anxiety agent and ending with the generated molecules not being in the input set. The bromine in bromazepam was replaced with chlorine in 16. This chlorination appears in other psychoactive drugs, lorazepam (17) and nordazepam (18). Also, the pyridine was replaced with chlorobenzene in 16. Both pyridine and chlorobenzene are aromatic, and chlorobenzene appears in clonazepam, another anti-anxiety drug.

## 4 Conclusion

We presented a molecule generator with two steps, which (i) generates scaffolds with a VAE and (ii) decorates the scaffolds with our transformer. Our extensive empirical results demonstrated the competitive performances of our model against baselines. In particular, our transformer architecture allowed us to learn complex SMILES syntax without any expert knowledge like pre-defined rules (say, substrings and parse trees). Also, our two-step model could capture the chemical rules of transforming an initial carbon scaffold into meaningful molecules. Interesting future work would be to develop a method for a seamless combination of our two steps.

## Supplementary Material

btac814_Supplementary_Data

## Data Availability

The MOSES dataset used in this work is available at https://github.com/molecularsets/moses. The ZINC-250K dataset is available at https://github.com/aspuru-guzik-group/chemical_vae.
