# Supplementary material for: Sc2Mol: a scaffold-based two-step molecule generator with variational autoencoder and transformer
Source: Bioinformatics. 2022 Dec 28;39(1):btac814. doi: 10.1093/bioinformatics/btac814 (PMC9835482; doi:10.1093/bioinformatics/btac814)
Supplement: btac814_Supplementary_Data [file btac814_supplementary_data.pdf]

Sc2Mol: A Scaffold-based Two-step Molecule Generator with Variational Autoencoder and Transformer

Supplementary information

Zhirui Liao Lei Xie, Hiroshi Mamitsuka, and Shanfeng Zhu

Table S1: Hyper parameter settings.

Hyper parameter	Value(s)
Batch size	64
# VAE encoder layers	3
# VAE decoder layers	3
VAE kernel size	3
# transformer layers	3
Latent variable dimension	64
Embedding dimension	256
Feed-forward dimension	1024
Dropout rate	0.1
KL-weight warm-up steps	40000
KL-weight start	0
KL-weight end	0.01
KL-weight increasement steps	5000
KL-weight increasement	1e-4

Table S2: Performances under three dimension settings (↑: Higher is better). Validity-check component was removed.

(d, d_z, d_{ff})	Validity↑	Uniqueness↑	Novelty↑
(128, 32, 512)	58.44%	99.97%	98.68%
(256, 64, 1024)	**63.07%**	**99.98%**	**98.70%**
(512, 128, 2048)	62.76%	99.97%	98.08%

Table S3: Performances under different # layers (↑: Higher is better). Validity-check component was removed.

# layers	Validity↑	Uniqueness↑	Novelty↑
1	43.16%	99.89%	98.29%
2	55.25%	**99.98%**	98.64%
3	63.07%	**99.98%**	**98.70%**
4	**64.98%**	99.96%	98.28%

Table S4: Performances of models for Task 1 on the ZINC dataset.

Model	Validity↑	Uniqueness↑	Novelty↑
AddCarbon	-	-	-
CharVAE	2.18%	73.85%	49.23%
FragLinker	-	-	-
JTVAE	**100.00%**	99.88%	99.58%
MoFlow	**100.00%**	99.87%	99.67%
Sc2Mol (Ours)	**100.00%**	**99.99%**	**99.97%**

↑: Higher is better

Table S5: Ablation study for Task 1 on ZINC.

Model	Validity↑	Uniqueness↑	Novelty↑
MoFlow	**100.00%**	99.87%	99.67%
MoFlow w.o. VC	31.76%	99.53%	99.61%
Sc2Mol	**100.00**%	**99.99%**	**99.97%**
Sc2Mol w.o. VC	59.46%	99.94%	98.85%

"w.o. VC" indicates "without validity-check component.

↑: Higher is better

Table S6: Benzodiazepine dataset for fine-tuning.

Name	SMILES
Bromazepam	BrC1=CC2=C(C=C1)NC(CN=C2C3=CC=CC=N3)=O
Camazepam	ClC1=CC2=C(C=C1)N(C)C(C(N=C2C3=CC=CC=C3)OC(N(C)C)=O)=O
Chlordiazepoxide	ClC1=CC2=C(N=C(NC)C[N+]([O-])=C2C3=CC=CC=C3)C=C1
Cinolazepam	FC1=CC=CC=C1C2=NC(C(N(CCC#N)C3=C2C=C(C=C3)Cl)=O)O
Clonazepam	[O-][N+](C1=CC2=C(C=C1)NC(CN=C2C3=CC=CC=C3Cl)=O)=O
Clorazepate	C1=CC=C(C=C1)C2=NC(C(=O)NC3=C2C=C(C=C3)Cl)C(=O)O
Delorazepam	ClC1=CC=CC=C1C2=NCC(NC3=C2C=C(C=C3)Cl)=O
Diazepam	c1ccccc1C2=NCC(=O)N(C)c3ccc(Cl)cc23
Doxefazepam	FC1=CC=CC=C1C2=NC(C(N(CCO)C3=C2C=C(C=C3)Cl)=O)O
Ethyl-loflazepate	FC1=CC=CC=C1C2=NC(C(OCC)=O)C(NC3=C2C=C(C=C3)Cl)=O
Fludiazepam	O=C1CN=C(C2=CC=CC=C2F)C3=CC(Cl)=CC=C3N1C
Flunitrazepam	[O-][N+](C1=CC2=C(C=C1)N(C)C(CN=C2C3=CC=CC=C3F)=O)=O
Flurazepam	FC1=CC=CC=C1C2=NCC(N(CCN(CC)CC)C3=C2C=C(C=C3)Cl)=O
Flutoprazepam	FC1=CC=CC=C1C2=NCC(N(CC3CC3)C4=C2C=C(C=C4)Cl)=O
Halazepam	FC(F)(CN1C(CN=C(C2=CC=CC=C2)C3=C1C=CC(Cl)=C3)=O)F
Ketazolam	ClC1=CC2=C(N(C)C(CN3C(C=C(C)OC32C4=CC=CC=C4)=O)=O)C=C1
Lorazepam	ClC1=CC=CC=C1C2=NC(C(NC3=C2C=C(C=C3)Cl)=O)O
Lormetazepam	ClC1=CC=CC=C1C2=NC(C(N(C)C3=C2C=C(C=C3)Cl)=O)O
Medazepam	ClC1=CC(C(C2=CC=CC=C2)=NCCN3C)=C3C=C1
Metaclazepam	ClC1=CC=CC=C1C2=NCC(COC)N(C)C3=C2C=C(Br)C=C3
Nimetazepam	[O-][N+](C1=CC2=C(C=C1)N(C)C(CN=C2C3=CC=CC=C3)=O)=O
Nitrazepam	[O-][N+](C1=CC2=C(C=C1)NC(CN=C2C3=CC=CC=C3)=O)=O
Nordazepam	ClC1=CC2=C(C=C1)NC(CN=C2C3=CC=CC=C3)=O
Oxazepam	OC1N=C(C2=C(NC1=O)C=CC(Cl)=C2)C3=CC=CC=C3
Phenazepam	c1ccc(c(c1)C2=NCC(=O)Nc3c2cc(cc3)Br)Cl
Pinazepam	Clc3cc1c(N(C(=O)CN=C1c2ccccc2)CC#C)cc3
Prazepam	Clc4cc1c(N(C(=O)CN=C1c2ccccc2)CC3CC3)cc4
Quazepam	FC(F)(F)CN1C(=S)CN=C(c2cc(Cl)ccc12)c3ccccc3F
Temazepam	CN1C2=C(C(C3=CC=CC=C3)=NC(O)C1=O)C=C(Cl)C=C2
Tetrazepam	CN1C(=O)CN=C(C2=C1C=CC(=C2)Cl)C3=CCCCC3
Clobazam	ClC1=CC(N(C2=CC=CC=C2)C(CC(N3C)=O)=O)=C3C=C1
Adinazolam	Clc3cc2C(=NCc1nnc(n1c2cc3)CN(C)C)c4ccccc4
Alprazolam	ClC1=CC2=C(C=C1)N3C(C)=NN=C3CN=C2C4=CC=CC=C4
Triazolam	ClC1=CC=CC=C1C2=NCC3=NN=C(C)N3C4=CC=C(Cl)C=C42
Flumazenil	Fc(c1)ccc-2c1C(=O)N(C)Cc3n2cnc3C(=O)OCC
Midazolam	ClC1=CC=C2C(C(C3=CC=CC=C3F)=NCC4=CN=C(C)N42)=C1

* Continued on the next page

Table S6 – continued from the previous page

Name	SMILES
Loprazolam	[O-][N+](=O)c1cc4c(cc1)N2C(=O)C(N=C2CN=C4c3ccccc3Cl)=CN5CCN(C)CC5
Cloxazolam	Clc1ccccc1C42OCCN2CC(=O)Nc3c4cc(Cl)cc3
Flutazolam	Fc1ccccc1C42OCCN2CC(=O)N(c3c4cc(Cl)cc3)CCO
Haloxazolam	Fc1ccccc1C42OCCN2CC(=O)Nc3c4cc(Br)cc3
Mexazolam	Clc1ccccc1C42OCC(N2CC(=O)Nc3c4cc(Cl)cc3)C
Clotiazepam	ClC1=C(C2=NCC(N(C)C3=C2C=C(CC)S3)=O)C=CC=C1
Brotizolam	ClC1=CC=CC=C1C2=NCC3=NN=C(C)N3C4=C2C=C(Br)S4
Etizolam	ClC1=CC=CC=C1C2=NCC3=NN=C(C)N3C4=C2C=C(CC)S4
Rilmazafone	ClC1=C(C(C2=CC(Cl)=CC=C2N3C(CNC(CN)=O)=NC(C(N(C)C)=O)=N3)=O)C=CC=C1
Zolazepam	FC1=CC=CC=C1C2=NCC(N(C)C3=C2C(C)=NN3C)=O
